# Incidence and Characteristics of Adverse Events after COVID-19 Vaccination in a Population-Based Programme

**DOI:** 10.3390/vaccines10071111

**Published:** 2022-07-12

**Authors:** Laura Bonzano, Olivera Djuric, Pamela Mancuso, Lidia Fares, Raffaele Brancaccio, Marta Ottone, Eufemia Bisaccia, Massimo Vicentini, Alessia Cocconcelli, Alfonso Motolese, Rostyslav Boyko, Paolo Giorgi Rossi, Alberico Motolese

**Affiliations:** 1 Dermatology Unit, Azienda Unità Sanitaria Locale—IRCCS di Reggio Emilia, 42122 Reggio Emilia, Italy; laura.bonzano@ausl.re.it (L.B.); raffaele.brancaccio@ausl.re.it (R.B.); alessia.cocconcelli@ausl.re.it (A.C.); alberico.motolese@ausl.re.it (A.M.); 2 Epidemiology Unit, Azienda Unità Sanitaria Locale—IRCCS di Reggio Emilia, 42122 Reggio Emilia, Italy; pamela.mancuso@ausl.re.it (P.M.); marta.ottone@ausl.re.it (M.O.); massimo.vicentini@ausl.re.it (M.V.); paolo.giorgirossi@ausl.re.it (P.G.R.); 3 Centre for Environmental, Nutritional and Genetic Epidemiology (CREAGEN), Section of Public Health, Department of Biomedical, Metabolic and Neural Sciences, University of Modena and Reggio Emilia, 41125 Modena, Italy; 4 Pharmaceutical Department, Azienda Unità Sanitaria Locale—IRCCS di Reggio Emilia, 42122 Reggio Emilia, Italy; lidia.fares@ausl.re.it; 5 Public Health Unit, Azienda Unità Sanitaria Locale—IRCCS di Reggio Emilia, 42122 Reggio Emilia, Italy; eufemia.bisaccia@ausl.re.it; 6 Section of Dermatology, Department of Clinical and Experimental Medicine, University of Messina, 98125 Messina, Italy; alfonsomotolese93@gmail.com; 7 Postgraduate School of Allergy and Clinical Immunology, University of Modena and Reggio Emilia, 41125 Modena, Italy; rostyslavboyko@yahoo.it

**Keywords:** COVID-19 vaccine, adverse events, allergy, risk stratification

## Abstract

Compliance with vaccination is linked to its safety. In Italy, a plan to identify people who could be at an increased risk of adverse events (AEs) was defined so they could be vaccinated in a protected setting. We conducted an audit to describe the process of AE risk assessment and occurrence in the Reggio Emilia Province in Italy in people who received any of the four COVID-19 vaccines currently used in Italy. Incidence of AEs was calculated by dose and type of vaccine and type of setting (standard vs. protected). After 182,056 first doses were administered, 521 (0.3%) AEs were reported. Most of the AEs were non-serious (91.4%) and non-allergic (92.7%). The percentage of AEs was similar in both settings: 0.3% in the standard setting and 0.2% in the protected setting. However, the incidence of AEs was higher among those who had an allergist visit than among those who did not (IR 666.7 vs. 124.9). All deaths (1.6/100.000) occurred in standard settings and after the Pfizer and Moderna vaccines. The incidence of AEs was lower after the second dose (IR 286.2 vs. 190.3), except for mRNA vaccines, for which it was higher after the second dose (IR 169.8 vs. 251.8). Although vaccination in a protected medical setting could reassure patients with a history of allergies to be vaccinated, allergy history and other anamnestic information is not useful in predicting the risk of COVID-19 vaccine-related AEs in the general population.

## 1. Introduction

Despite initial hesitancy and mistrust in vaccine safety in Italy [1], the majority of the population has been vaccinated. As of the end of December 2021, 9.02 billion doses have been administered globally, with 57.4% of the world population receiving at least one dose of a COVID-19 vaccine [2]. Almost 90% of the Italian population has received at least one dose of the vaccine, with 84% of the eligible population fully vaccinated [3].

The first vaccine against SARS-CoV-2 approved by the European Medicines Agency and the Italian Medicines Agency (AIFA) and made available in Italy was the Comirnaty vaccine, developed by Pfizer-BioNTech. On 28 December 2020, the first doses were administered to healthcare workers. In January 2021, Moderna and Oxford-AstraZeneca vaccines were approved, followed by the approval of the Janssen vaccine on 11 March 2021.

The first reports of adverse events after vaccination were those identified by randomized control trials assessing their effectiveness and safety [4,5,6,7]. However, these studies only included healthy volunteers, excluding participants with a history of an allergic reactions or immunocompromising conditions.

Post-marketing surveillance of the vaccine effects in the real world is fundamental to ensure safety, to inform policy concerning mass vaccination, and to guide clinicians in individual vaccine recommendations. Data from real-world surveillance have been reported [8,9,10], confirming the overall safety profile but also identifying some excess risk of rare adverse events, in particular anaphylaxis, thrombosis with thrombocytopenia syndrome, stroke, some immune-mediated disorders such as Guillain-Barré Syndrome [11], and hormonal disorders [12]. Nevertheless, data from AE surveillance systems are affected by several biases and cannot quantify the real burden of mild adverse events and those for whom adverse events may be more common. There are several studies to date reporting adverse events from different perspectives or populations, neurological AEs [13], AEs requiring admissions to EDs [14], and AEs among HCWs [15,16,17]. However, there are few comprehensive population-based reports, especially stratified according to the risk of allergic reactions.

Compliance with vaccination is linked to its safety. The Centers for Disease Control and Prevention (CDC) published different recommendations regarding the safety of these vaccines for patients with a history of allergic reactions [18]. The Italian Society of Allergy, Asthma and Clinical Immunology (SIAAIC) recommends that people with known allergies and a history of anaphylaxis should not be excluded from vaccination; instead, they require a more specific and individualised management, such as prolonged observation, premedication, or stabilisation of the underlying disease. In addition, they should be vaccinated in an appropriately staffed setting with immediately available aids to deal with serious anaphylactic emergencies [19]. To ensure the highest level of safety possible and to facilitate a greater response to vaccination, Italy defined a plan to identify people who could be at an increased risk of adverse effects and to vaccinate them in a safe setting. Although the risk stratification was developed mainly for the purpose of assessing history of allergy, people with non-allergy conditions, such as malignancy, obesity, or frailty, were vaccinated in the protected setting if deemed necessary.

This audit report aims to describe the process of adverse effect risk assessment and the occurrence of allergic and non-allergic adverse effects in the population who underwent vaccination in the Reggio Emilia Province.

## 2. Materials and Methods

### 2.1. Study Design

Data on adverse events reported to AIFA were used to describe the characteristics and calculate the incidence of events in the cohort of people who received the first dose between 27 December 2020 and 12 September 2021 in the Reggio Emilia Province. Persons included were those who had a suggested exemption from a GP or an appointment or presented voluntarily for the first dose of any of the COVID-19 vaccines currently authorised and used in Italy: Pfizer–BioNTech’s Comirnaty (BNT162b2), Spikevax Moderna COVID-19 vaccine (mRNA-1273), Vaxzeveria AstraZeneca AZD1222 (ChAdOc1-S nCoV-19) and Johnson & Johnson’s Janssen (J&J/Janssen) COVID-19 vaccine (JNJ-78436735).

We report AEs following the first and second dose of the COVID-19 vaccine, both as a proportion of AEs out of the total AEs reported and from the total patients vaccinated.

### 2.2. Triage and Risk Stratification

According to the local vaccination protocol at the time, persons with low risk of AEs and persons with temporary exemption due to non-allergy indications (quarantine, current or previous COVID-19 infection, ongoing influenza, and pregnancy) were vaccinated in a standard setting, such as a fair hall, following standard procedures that included waiting for 15 min after vaccination, with all the necessary aids and drugs available and medical staff able to recognise and manage possible allergic reactions. Persons with suspected allergies, persons with a history of allergies that have never resulted in anaphylaxis (to food, pollen, insect bites and stings) with chronic urticarial or cutaneous reactions to non-official drugs, persons with a positive family history of reactions to drugs or patients with a history of severe allergic reactions (e.g., anaphylaxis) to any antigen except injectable drugs and vaccines were considered to be at moderate/high risk and were referred to an allergy evaluation. Those without confirmed cutaneous allergic reactions to drugs that contain PEG were vaccinated in a protected setting. The protected setting could be either an outpatient or a hospital setting, with easy access to the recovery room and with the immediate availability of medical devices to deal with serious anaphylactic emergencies and allowing a stay of 30 min for observation after vaccination.

A general questionnaire was sent to all persons scheduled for vaccination via phone message or email, either before the vaccination session or when arriving at the vaccination site. Those who answered positively to one of the allergy-related questions were asked four more specific questions once they presented for the vaccination session. They were asked by the vaccinating doctor: (1) if they suffer from severe anaphylaxis (involvement of the cardiovascular and/or respiratory system) from any cause or an unknown cause; (2) if they suffer from uncontrolled asthma; (3) if they were diagnosed with mastocytosis; (4) if they have had allergic reactions to any drug or previous vaccinations or to polyethylene glycol (PEG) and polysorbates. If the answer was “no” to all four questions, the individual was considered “low risk” and proceeded to the vaccination under the usual protocol with a 15 min observation period. Patients with at least one of these conditions were considered at moderate/high risk and were referred to an allergy appointment to carry out specific diagnostics. The public health department of the local health authority receives these requests first and evaluates whether they could be vaccinated or should be referred to an allergy evaluation. In addition to doctors from the vaccination centre, the public health department can also receive and evaluate requests from general practitioners, paediatricians, and dermatologists. Furthermore, general practitioners and specialists could notify the public health department of patients with conditions or diseases that may qualify them for inclusion in the high-risk pathway or exempt them from vaccination temporarily or permanently. Therefore, some persons with non-allergy conditions, such as malignancy, obesity, or frailty, were vaccinated in the protected setting if deemed necessary. The public health department examined all reports and made decisions on whether an allergy appointment is needed and if and where people could receive the vaccination.

Allergy evaluations were done before the first dose, and in the case of suspected reactions after the first dose, they could be done before the second dose. They included cutaneous tests if indicated to evaluate possible COVID-19 vaccine hypersensitivity, such as COVID-19 vaccine excipients, specifically polyethylene glycol (PEG), polysorbate 80, and polysorbate 20.

Persons with allergies to drugs that contain PEG (Depomedrol, Depoprovera, SonoVue, Herzeptin/Herzuma/Trastuzumab, Arcalyst, Definity, Zirtec, Taxotere, Neulasta, Mircrea/Epoetin beta, etc.) or polysorbate (Amiodarone, Taxotere, Lantus, Alpidra, Trulicity, Xolari, Herceptin/Herzuma, Invega, Humira, Tremfya, Remsima, monoclonal antibodies, Kenakort, growth factors, etc.), confirmed by skin tests were exempted from vaccination.

### 2.3. Triage for the Second Dose

In the case of a reaction to the first dose of the COVID-19 vaccine, the vaccination with the second dose depended on the extent of the reaction. In the case of local or mild general symptoms, the vaccine could be administered in a standard setting. If the patient had vaso-vagal crisis, tremor, tingle, or anxiety after the first dose, the observation period could be 30 min in the standard setting. In the case of the presence of local symptoms that were particularly important, such as a wheal larger than 10 cm or a late urticarial reaction that started 60 min after the first dose, the vaccine could be administered in the standard setting but with Loratidin or Deltacortene 1 h before the second dose. If the reaction after the first dose was an immediate allergic reaction occurring within 60 min of vaccination, such as generalised urticaria, angioedema of larynx, eyes, or lips, dyspnea, chest tightness, hypotension, gastrointestinal symptoms, or unconsciousness, the vaccination was suspended, and an allergy evaluation was considered. In the case of anaphylaxis after the first dose of mRNA vaccine or any of its components, or in the case of infection with SARS-CoV-2 after the first dose, the second dose was not administered.

### 2.4. Reporting of AEs

Adverse drug reactions (ADRs) were reported to the Pharmacovigilance Service in the form of Individual Case Safety Reports (ICSRs) by the general practitioner/family doctor, the Vaccination Centre, the pharmacist, or the Local Health Authority (ASL) or directly by the patient by filling in the form available on the AIFA portal or through the VigiFarmaco application. The severity category of AEs is selected by the reporter during the compilation of the safety form following the indications available on the portal. Cases that do not comply with the criteria for AE or contain missing information are controlled by the regional health authority and AIFA.

### 2.5. Data Sources

Data about vaccination appointments, vaccines administered, and persons exonerated were retrieved from the local health authority vaccine information system. Data about allergy testing and test results were obtained from the allergy database of the dermatology unit. Adverse events data were obtained from the pharmaceutical department of the local health authority.

### 2.6. Definition of Adverse Events

Serious AEs considered 75 different clinically serious conditions divided into five categories according to the recommendations of AIFA [20]: hospitalisation or prolonged hospitalisation, severe or permanent invalidity, life-threatening conditions, death, and other clinically relevant conditions.

### 2.7. Statistical Analysis

Descriptive statistics were used, and data are presented as frequency and percentages. Incidence rate (IR) was calculated as the number of AEs per 100,000 persons vaccinated. Analyses were stratified by vaccine dose, risk pathway, and type of vaccine. STATA 16.1 (Stata Corporation, College Station, TX, USA) was used for all analyses.

## 3. Results

### 3.1. First Dose

**Figure 1:**
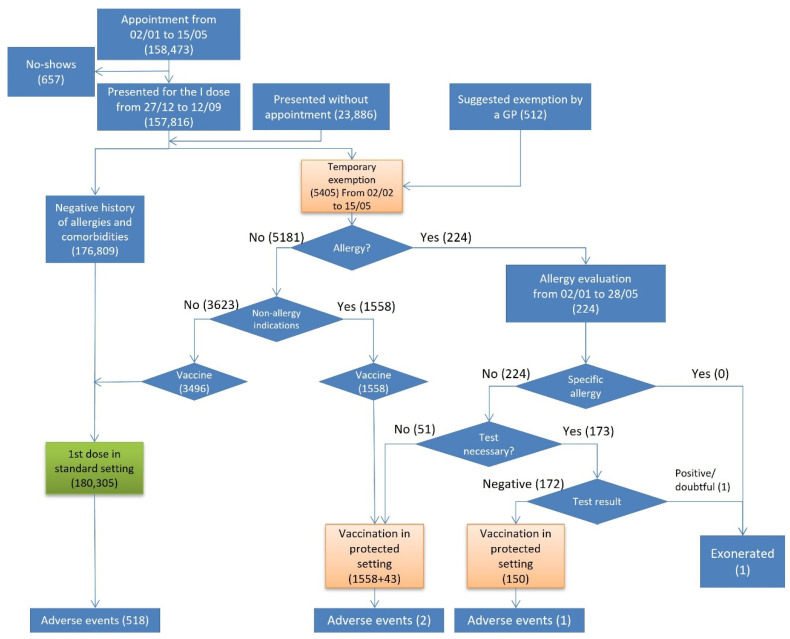
Flowchart of the first dose vaccinations, exonerations, and adverse events by risk pathway.

Out of 182,214 persons who presented for vaccination, 180,305 persons were vaccinated in a standard setting (green pathway), 1751 were vaccinated in the protected setting, 1 person was exonerated, and 157 did not get vaccinated (Figure 1).

Out of 5405 who were temporarily exempted, 224 reported histories of an allergy and were referred to an allergy evaluation. While no one had a vaccine-specific allergy, 173 undertook an allergy test. Only one person was exonerated from the vaccination, since he demonstrated inconclusive skin test results for COVID-19 vaccine excipients. Out of 5181 persons who were temporarily exempted due to non-allergy indications, 1558 were vaccinated in the protected setting.

Overall, 521 (0.3%) AEs were reported after 182,056 first doses administered, out of which 281 were self-reported (53.9%) and 240 reported by a health worker (46.1%) (Table 1). Out of 180,305 persons vaccinated in the standard setting, 518 (0.3%) had an adverse event (Figure 1). In the protected setting, 3 (0.2%) out of 1751 vaccinated persons developed an adverse event, all of which were non-severe, and one occurred in a person with a history of allergy. The incidence of AEs was higher among those who had been referred to an allergy evaluation than among those who did not (IR 666.7 vs. 124.9) (Table 2).

Although 85.4% (445/521) of AEs were systemic, most of them were non-serious (91.4%) (476/521) and non-allergic (92.7%) (483/521) (Table 1). All serious AEs happened in the standard setting. The list of specific conditions reported as severe and categorized by organ system and dose is summarized in Supplementary Table S1. Death occurred in 1.6/100.000 persons. All deaths occurred in the standard setting and after the Pfizer and Moderna vaccines (Supplementary Table S2).

AEs were mostly reported by females (73.1%, IR 369.3 vs. 177.5) during the first 24 h after vaccination (78.5%) in the age category 50–59 years (IR 418.6) (Table 1) and after administration of the AZ/JJ vaccine (59.3%, IR 540.2 vs. 169.8) (Supplementary Table S3).

### 3.2. Second Dose

**Figure 2:**
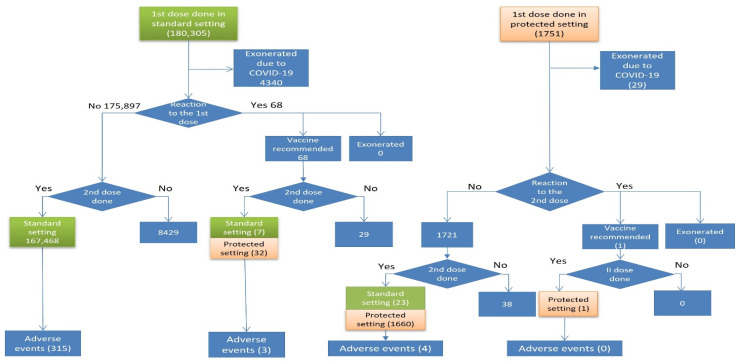
Flowchart of the second dose vaccinations, exonerations, and adverse events by risk pathway.

Out of 180,305 persons vaccinated with the first dose in the standard setting, 4340 (2.4%) were temporarily exempted due to various reasons including positivity to COVID-19 or being isolated as a contact of a positive person (Figure 2). Out of 68 persons who had an adverse reaction after the first dose and were eligible for the second dose, 32 were vaccinated in the protected setting and seven in the standard setting. Out of 1751 persons vaccinated with the first dose in the protected setting, 29 (1.7%) were exonerated due to positivity to COVID-19. One person who had an adverse event after the first dose was vaccinated in the protected setting with no adverse events after the second dose.

Overall, 169,191 persons were vaccinated with the second dose: 167,498 in the standard setting and 1693 in the protected setting. After the second dose, 322 (0.2%) AEs were reported: 133 (41.3%) self-reported and 189 (58.7%) reported by a healthcare worker (Table 1). Out of 322 AEs reported, 315 (97.8%) occurred in the standard setting and 7 (2.2%) in the protected setting (Table 3). The incidence of AEs was lower after the second dose (IR 286.2 vs. 190.3), while the incidence of death was higher after the second dose (2.4/100,000 vs. 1.6/100,000) if the period after vaccination was unrestricted. Restricting the period after vaccination to 21 days, the incidence of death became similar for both doses (1.6 vs. 1.8). All four persons who died after the second dose didn’t have any AE after the first dose.

Although the overall incidence of AEs after the second dose was the highest in persons who had a reaction after the first dose but were vaccinated in the standard setting (IR 7692.3), all 27 severe AEs after the second dose occurred in people who did not report an AE after the first dose (Table 3). In percentages, there were 8.6% of severe AEs after the first dose, 8.4% after the send dose overall, and 8.5% after the second and not after the first dose.

Similar to the first dose, most of the AEs were not severe (91.6%), non-allergic (93.8%), immediate (84.8%), and systemic (88.2%). All severe and 99% of non-severe AEs after the second dose occurred in people who had not had an AE after the first dose. In contrast to the first dose, a substantially higher incidence of AEs was associated with the second dose of Pfizer/Moderna than with AZ/JJ (IR 151.8 vs. 53.4) (Supplementary Tables S4 and S5).

## 4. Discussion

The occurrence of AEs in our study was similar in both settings, suggesting that the process put in place to identify a person’s increased risk of AEs was not effective. Furthermore, the specialist allergy evaluations identified only one person who should be exonerated due to a confirmed allergy to PEG. The only group that showed an increased risk of AEs was that including those who had a reaction to the first dose. However, these reactions were mostly non-severe and non-systemic; almost all non-severe and severe AEs (including death) after the second dose occurred in persons who did not have an adverse reaction to the first dose.

There are still many uncertainties regarding whether or not persons with a history of allergies or anaphylaxis should be vaccinated with a COVID-19 vaccine. When considering only allergic AEs, we found 0.001% of allergic reactions after the first dose and 0.002% after the second dose. The Israeli study on immunisation with the BNT162b2, the only study so far to evaluate allergic reactions in highly allergic individuals, reported nearly 2% of allergic reactions (1.4% after the first dose and 1.8% after the second dose) [21]. However, they included only 429 persons with a high risk of allergies who were referred to the allergy evaluation and vaccinated in supervised medical settings, with no comparison to the population vaccinated in standard settings. Thus, the small sample size might be a reason for a much higher rate of allergic AEs in their study. Another reason could be the fact that in our study persons with non-allergy indications (n = 1558 for the first dose) were vaccinated in the protected setting as well, which could have diluted the rate of allergic AEs.

Our results, in terms of share of non-serious AEs, death, sex distribution, HCW-reported AEs, and occurrence within 48 h, are very similar to the results of the Italian Medicines Agency (Agenzia Italiana del farmaco-AIFA) report for the entirety of Italy [22]. However, overall percentage and incidence were lower in the AIFA report than in our study for both doses (dose I: 158 vs. 286.2–100,000 doses; dose II: 77 vs. 190.3/100,000 doses). According to their estimation, the majority were in the category “other clinically relevant conditions”, and for only around one-third (36%) of the reported severe AEs a causal link with the vaccine could be confirmed.

The overall incidence of AEs and serious AEs in our study was lower after the second dose, but for mRNA vaccines Pfizer and Moderna, it was higher after the second dose. Similar findings were found by the CDC’s studies and in clinical trials in which higher reactogenicity was reported after the second dose of Pfizer-BioNTech [23,24]. However, the incidence of death was similar after both doses in our study. All seven deaths reported (three after the first dose and four after the second dose) in our study were reported after the Comirnaty vaccine. It is unlikely that these deaths were a consequence of anaphylaxis but it is more likely that they were due to the worsening of an underlying condition [22]. This is also supported by the fact that they all occurred in persons vaccinated in the standard setting among persons with no reactions reported after the first dose.

A common finding to date is that an AE is disproportionately reported by women. This was also the case in our study (73.1 and 74.5% of AEs after the first and second dose, respectively, were reported by women) and in previous reports and vaccines [8,23,25]. Although behavioural factors such as lower reporting by men due to perceived self-rated health probably play an important role in disproportional AE incidence between sexes, various genetic, hormonal, and biological determinants are considered fundamental for the robustness of female immune response to both their own and foreign antibodies [26,27,28]. Further studies are needed to define exact social, demographic, and clinical risk factors for allergic AEs following COVID-19 vaccination.

The major limitation of our study is that the AEs for the second dose were self-reported or reported by healthcare workers and not actively and systematically collected, while for the first dose, an active collection of all reactions was done when people presented for the second dose. Individual case reports by the multidisciplinary expert team and standardized causality assessment algorithms should be applied for both doses in order to improve AE reporting. Due to the descriptive design of the study and lack of official test to estimate the effectiveness of the risk stratification, it is not possible to make any causal inference. However, this is the first population-based study to evaluate the risk of various AEs according to the allergy risk stratification; we defined a cohort that included all the people who presented for vaccination, and we collected all the referrals and procedures for assessing AE risk. Although it might be generalizable only to the settings with similar organizational and epidemiological contexts, this audit might be useful to inform future vaccination strategies in patients at risk of AEs or those who have already experienced an AE.

## 5. Conclusions

The percentage and incidence of AEs after all COVID-19 vaccines currently approved and used in Italy is very low, and most AEs have a local, non-severe character. Despite a large effort in screening and assessing the risk of AEs, the occurrence of AEs was similar in all risk-stratified groups. Only reactions after the first dose identified a higher risk of AEs, but this stratification only identified a small proportion of AEs in second doses, which were mostly non-severe cases. Although vaccination in a protected, medical setting could calm and reassure patients to be vaccinated, a history of allergies and non-allergic comorbidities is not useful in predicting the risk of COVID-19 vaccine-related AEs in the general population.

## Figures and Tables

**Table 1:** Total number of AEs after first and second dose.

	Dose I			Dose II		
	N of AEs	N of Vaccinated	Incidence (95% CI)	N of AEs	N of Vaccinated	Incidence (95% CI)
Overall	521	182,056	286.2 (262.2–311.8)	322	169,191	190.3 (170.1–212.3)
Severity						
Severe	45	182,056	24.7 (18.0–33.1)	27	169,191	16.0 (10.5–23.2)
Other clinically relevant condition	20			11		
Death	3			4		
Severe or permanent disability	0			1		
(Prolonged) hospitalisation	20			5		
Life-threatening condition	2			6		
Not severe	476	182,056	261.5 (238.5–286.0)	295	169,191	174.4 (155.0–195.4)
Onset time						
Immediate (≤24 h)	409	182,056	224.7 (203.4–247.5)	273	169,191	161.4 (142.8–181.7)
Non-immediate (>24 h)	112	182,056	61.5 (50.7–74.0)	49	169,191	29.0 (21.4–38.3)
Local vs. systemic AE						
Local	36	182,056	19.8 (13.9–27.4)	14	169,191	8.3 (4.5–13.9)
Systemic	445	182,056	244.4 (222.3–268.2)	284	169,191	167.9 (148.9–188.5)
Insufficient information	40	182,056	22.0 (15.7–29.9)	24	169,191	14.2 (9.1–21.1)
Allergic vs. not allergic AE						
Allergic	2	182,056	1.1 (0.1–4.0)	4	169,191	2.4 (0.6–6.1)
Not allergic	483	182,056	265.3 (242.2–290.0)	302	169,191	178.5 (159.0–199.8)
Doubtful	33	182,056	18.1 (12.5–25.5)	12	169,191	7.1 (3.7–12.4)
Insufficient information	3	182,056	1.7 (0.3–4.8)	4	169,191	2.4 (0.6–6.1)
Type of reporter						
Self-reported	281	182,056	154.4 (136.8–173.5)	133	169,191	78.6 (65.8–93.2)
Health worker	240	182,056	131.8 (115.7–149.6)	189	169,191	111.7 (96.4–128.8)
Sex						
Male	140	78,883	177.5 (149.3–209.4)	82	73,230	112.0 (89.1–139.0)
Female	381	103,176	369.3 (333.2–408.2)	240	95,961	250.1 (219.5–283.8)
Age						
<50	219	37,849	578.6 (504.7–660.3)	162	35,144	461.0 (392.8–537.5)
50–59	104	24,844	418.6 (342.2–507.0)	73	23,094	316.1 (247.9–397.3)
60–69	84	41,101	204.4 (163.1–253.0)	39	36,375	107.2 (76.3–146.5)
70–79	85	43,174	196.9 (157.3–243.4)	22	40,843	53.9 (33.8–81.5)
80+	29	35,088	82.6 (55.4–118.7)	26	33,735	77.1 (50.4–112.9)
Vaccine type						
Pfizer/Moderna	212	124,852	169.8 (147.7–194.2)	294	116,738	251.8 (223.9–828.3)
AZ/JJ	309	57,204	540.2 (481.8–603.7)	28	52,453	53.4 (35.5–77.1)
Vaccination motive						
Population	184	97,711	188.3 (162.1–217.5)	63	91,652	68.7 (52.8–87.9)
Comorbidity	58	45,349	127.9 (97.1–165.3)	71	41,032	173.0 (135.2–218.2)
Long-term care facility (LTCF)	4	3148	127.1 (34.6–325.0)	2	2669	74.9 (9.1–270.4)
Work	275	35,848	767.1 (679.4–863.0)	186	33,838	549.7 (473.7–634.3)
Previous SARS-CoV-2 infection						
No	485	173,444	279.6 (255.3–305.6)	312	165,468	188.6 (168.2–210.7)
Yes	36	8612	418.0 (292.9–578.3)	10	3723	268.6 (128.9–493.4)

**Table 2:** Characteristics of adverse events after the first dose of COVID-19 vaccine, by risk pathway.

	Dose I								
	Green Pathway			Yellow Pathway without Allergology Visit			Yellow Pathway with Allergology Visit		
	N of AEs	N of Vaccinated	Incidence (95% CI)	N of AEs	N of Vaccinated	Incidence (95% CI)	N of AEs	N of Vaccinated	Incidence (95% CI)
Overall	518	180,305	287.3 (263.1–313.1)	2	1601	124.9 (15.1–450.5)	1	150	666.7 (16.9–3658.3)
Severity									
Severe	45	180,305	25.0 (18.2–33.4)	0	1601	0	0	150	0
Other clinically relevant condition	20			0			0		
Death	3			0			0		
Severe or permanent disability	0			0			0		
(Prolonged) hospitalisation	20			0			0		
Life threatening condition	2			0			0		
Not severe	473	180,305	262.3 (239.3–287.0)	2	1601	124.9 (15.1–450.5)	1	150	666.7 (16.9–3658.3)
Onset time									
Immediate (≤24 h)	407	180,305	225.7 (204.4–248.7)	1	1601	62.5 (1.6–347.5)	1	150	666.7 (16.9–3658.3)
Non-immediate (>24 h)	111	180,305	61.6 (50.7–74.1)	1	1601	62.5 (1.6–347.5)	0	150	0
Local vs. systemic AE									
Local	36	180,305	20.0 (14.0–27.6)	0	1601	0	0	150	0
Systemic	442	180,305	245.1 (222.8–269.1)	2	1601	124.9 (15.1–450.5)	1	150	666.7 (16.9–3658.3)
Insufficient information	40	180,305	22.2 (15.9–30.2)	0	1601	0	0	150	0
Allergic vs. not allergic AE									
Allergic	2	180,305	1.1 (0.1–4.0)	0	1601	0	0	150	0
Not allergic	481	180,305	266.8 (243.5–291.7)	1	1601	62.5 (1.6–347.5)	1	150	666.7 (16.9–3658.3)
Doubtful	32	180,305	17.8 (12.1–25.1)	1	1601	62.5 (1.6–347.5)	0	150	0
Insufficient information	3	180,305	1.7 (0.3–4.9)	0	1601	0	0	150	0
Type of reporter									
Self-reported	279	180,305	154.7 (137.1–174.0)	1	1601	62.5 (1.6–347.5)	1	150	666.7 (16.9–3658.3)
Health worker	239	180,305	132.6 (116.3–150.5)	1	1601	62.5 (1.6–347.5)	0	150	0
Sex									
Male	139	78,299	177.5 (149.3–209.5)	1	565	177.0 (4.5–982.2)	0	19	0
Female	379	102,006	371.5 (335.1–410.8)	1	1039	96.2 (2.4–535.1)	1	131	763.4 (19.3–4179.7)
Age									
<50	219	37,690	581.1 (506.8–663.1)	0	127	0	0	32	0
50–59	103	24,671	417.5 (340.9–506.1)	0	141	0	1	32	3125.0 (79.1–16,217.1)
60–69	83	40,837	203.2 (161.9–251.9)	1	240	416.7 (10.6–2299.5)	0	24	0
70–79	84	42,130	199.4 (159.1–246.8)	1	1012	98.8 (2.5–549.3)	0	32	0
80+	29	34,977	82.9 (55.5–119.1)	0	81	0	0	30	0
**Vaccine type**									
Pfizer/Moderna	209	123,101	169.8 (147.6–194.4)	2	1601	124.9 (15.1–450.5)	1	150	666.7 (16.9–3658.3)
AZ/JJ	309	57,204	540.2 (481.8–603.7)	0	0	-	0	0	-
**Vaccination motive**									
Population	183	97,168	188.3 (162.1–217.7)	1	475	210.5 (5.3–1167.4)	0	68	0
Comorbidity	57	44,273	128.7 (97.5–166.8)	1	1027	97.4 (2.5–541.3)	0	49	0
Long-term care facility (LTCF)	4	3147	127.1 (34.6–325.1)	0	1	0	0	0	-
Work	274	35,717	767.1 (679.3–863.2)	0	98	0	1	33	3030.3 (76.7–15,759.4)

**Table 3:** Characteristics of adverse events after the second dose of COVID-19 vaccine, by risk pathway.

	Dose II											
	Green Pathway						Yellow Pathway					
	Reaction to Dose I			No Reaction to Dose I			Reaction to Dose I			No Reaction to Dose I		
	N of AEs	N of Vaccinated	Incidence (95% CI)	N of AEs	N of Vaccinated	Incidence (95% CI)	N of AEs	N of Vaccinated	Incidence (95% CI)	N of AEs	N of Vaccinated	Incidence (95% CI)
Overall	3	39	7692.3 (1615.3–20,870.2)	315	167,468	188.1 (167.9–210.0)	0	1	0	4	1683	237.7 (64.8–607.4)
Severity												
Severe	0			26	167,468	15.5 (10.1–22.8)	0			1	1683	59.4 (1.5–330.6)
Other clinically relevant condition	0			11			0			0		
Death	0			4			0			0		
Severe or permanent disability	0			1			0			0		
(Prolonged) hospitalisation	0			5			0			0		
Life threatening condition	0			5			0			1		
Not severe	3	39	7692.3 (1615.3–20,870.2)	289	167,468	172.6 (153.3–193.6)	0	1	0	3	1683	178.3 (36.8–520.0)
Onset time												
Immediate (≤24 h)	2	39	5128.2 (672.2–17,324.5)	267	167,468	159.4 (140.9–179.7)	0	1	0	4	1683	237.7 (64.8–607.4)
Non-immediate (>24 h)	1	39	2564.1 (64.9–13,476.4)	48	167,468	28.7 (21.1–38.0)	0	1	0	0	1683	0
Local vs. systemic AE												
Local	0	39	0	14	167,468	8.4 (4.6–14.0)	0	1	0	0	1683	0
Systemic	3	39	7692.3 (1615.3–20,870.2	277	167,468	165.4 (146.5–186.1)	0	1	0	4	1683	237.7 (64.8–607.4)
Insufficient information	0	39	0	24	167,468	14.3 (9.2–21.3)	0	1	0	0	1683	0
Allergic vs. not allergic reactions												
Allergic	0	39	0	4	167,468	2.4 (0.7–6.1)	0	1	0	0	1683	0
Not allergic	3	39	7692.3 (1615.3–20,870.2	296	167,468	176.8 (157.2–198.1)	0	1	0	3	1683	178.3 (36.8–520.0)
Doubtful	0	39	0	11	167,468	6.6 (3.3–11.8)	0	1	0	1	1683	59.4 (1.5–330.6)
Insufficient information	0	39	0	4	167,468	2.4 (0.7–6.1)	0	1	0	0	1683	0
Type of reporter												
Self-reported	0	39	0	132	167,468	78.8 (66.0–93.5)	0	1	0	1	1683	59.4 (1.5–330.6)
Healthcare worker	3	39	7692.3 (1615.3–20,870.2	183	167,468	109.3 (94.0–126.3)	0	1	0	3	1683	178.3 (36.8–520.0)
Sex												
Male	0	3	0	80	72,664	110.1 (87.3–137.0)	0	0	-	2	563	355.2 (43.1–1277.3)
Female	3	36	8333.3 (1752.7–22,469.0)	235	94,804	247.9 (217.2–281.6)	0	1	0	2	1120	178.6 (21.6–643.6)
Age												
<50	1	15	6666.7 (168.6–31,948.5)	160	34,982	457.4 (389.4–533.8)	0	0	-	1	147	680.3 (17.2–3731.8)
50–59	2	13	15,384.6 (1920.7–45,447.1)	69	22,920	301.1 (234.3–380.8)	0	1	0	2	160	1250.0 (151.7–4442.3)
60–69	0	2	0	39	36,123	108.0 (76.8–147.6)	0	0	-	0	250	0
70–79	0	6	0	21	39,813	52.7 (32.7–80.6)	0	0	-	1	1024	97.7 (2.5–542.9)
80+	0	3	0	26	33,630	77.3 (50.5–113.3)	0	0	-	0	102	0
Vaccine type												
Pfizer/Moderna	3	34	8823.5 (1858.0–23,677.5)	287	115,020	249.5 (221.5–280.1)	0	1	0	4	1683	237.7 (64.8–607.4)
AZ/JJ	0	5	0	28	52,448	53.4 (35.5–77.2)	0	0	-	0	0	-
Vaccination motive												
Population	0	9	0	62	91,127	68.0 (52.2–87.2)	0	0	-	1	516	193.8 (4.9–1075.0)
Comorbidity	0	4	0	70	39,981	175.1 (136.5–221.2)	0	0	-	1	1047	95.5 (2.4–531.0)
Long-term care facility (LTCF)	0	0	-	2	2668	75.0 (9.1–270.5)	0	0	-	0	1	0
Work	3	26	11,538.5 (2445.8–30,154.0)	181	33692	537.2 (462.0–621.2)	0	1	0	2	119	1680.7 (204.2–5939.3)

## Data Availability

Data are available upon reasonable request. According to Italian law, anonymized data can only be made publicly available if there is no potential for the re-identification of individuals (https://www.garanteprivacy.it; accessed on 12 July 2022). Thus, the data underlying this study are available on request to researchers who meet the criteria for access to confidential data. In order to obtain data, approval must be obtained from the Area Vasta Emilia Nord (AVEN) Ethics Committee, who would then authorize us to provide aggregated or anonymized data. Data access requests should be addressed to the Ethics Committee at CEReggioemilia@ausl.re.it as well as to the authors at the Epidemiology unit of AUSL–IRCCS of Reggio Emilia at info.epi@ausl.re.it, who are the data guardians.

## Supplementary Materials

The following supporting information can be downloaded at: https://www.mdpi.com/article/10.3390/vaccines10071111/s1, Table S1: List of severe AEs by organ system and dose; Table S2: Characteristics of adverse events after the first dose of Pfizer/Moderna COVID-19 vaccine; Table S3: Characteristics of adverse events after the first dose of AstraZeneca and J&J/Janssen COVID-19 vaccine; Table S4: Characteristics of adverse events after the second dose of Pfizer/Moderna COVID-19 vaccine; Table S5: Characteristics of adverse events after the second dose of AstraZeneca and J&J/Janssen COVID-19 vaccine.
