# Supplementary material for: Incidence and Characteristics of Adverse Events after COVID-19 Vaccination in a Population-Based Programme
Source: Vaccines (Basel). 2022 Jul 12;10(7):1111. doi: 10.3390/vaccines10071111 (PMC9324067; doi:10.3390/vaccines10071111)
Supplement: Supplementary file 1 [file vaccines-10-01111-s001.zip › vaccines-1745941-supplementary.pdf]

Supplementary material

Table S1. List of severe AEs by organ system and dose

	Total	1st dose	2nd dose
**General**	20	14	6
Headache, fever, and arthralgia	17	13	4
Syncope	4	2	2
**Cardiological AE**	6	4	2
Myocarditis	1	1	0
Pericarditis	1	0	1
Acute myocardial infarction	1	1	0
Arrhythmias	3	2	1
**Vascular disorders**	11	6	5
Thromboembolism	8	4	4
Vascular diseases of the legs	3	2	1
**Nervous system disorders**	9	6	3
Stroke	3	1	2
Epileptic seizure	1	1	0
Bell's palsy	1	1	0
Aphasia	1	1	0
Upper extremity paraesthesia	1	0	1
Paresthesia, vertigo, vomiting, postural instability	1	1	0
Cranial nerve palsy	1	1	0
**Blood and lymphatic system disorders**	4	3	1
Thrombocytopenia	4	3	1
Lymphadenopathy	1	0	1
**Allergy (angioedema, swelling of the face, etc)**	3	2	1
**ARDS**	4	2	2
**Pancreatitis**	1	0	1
**Meningoencephalitis**	1	1	0

**Still's Disease**	1	1	0
**Sudden death**	7	3	4
**Other**	2	1	1
Fall	1	0	1
Q fever	1	1	0
Total	72	45	27

Table S2. Characteristics of adverse events after the first dose of Pfizer/Moderna COVID-19 vaccine.

	I Dose											
	Total			Green pathway			Yellow pathway without allergology visit			Percorso giallo con visita allergologica		
	N of AEs	N of vaccinated	Incidence	N of AEs	N of vaccinated	Incidence	N of AEs	N of vaccinated	Incidence	N of AEs	N of vaccinated	Incidence
**Overall**	212	124852	169.8	209	123101	169.8	2	1601	124.9	1	150	666.7
**Severity**												
Severe	19			19			0			0		
Other clinically relevant condition	*8*			*8*			*0*			*0*		
Death	*3*			*3*			*0*			*0*		
Severe or permanent disability	*0*			*0*			*0*			*0*		
(Prolonged) hospitalisation	*6*			*6*			*0*			*0*		
Life threatening condition	*2*			*2*			*0*			*0*		
Not severe	193			190			2			1		
**Onset time**												
Immediate (≤ 24h)	147			145			1			1		
Non-immediate (> 24h)	65			64			1			0		
**Local vs systemic AE**												
Local	29			29			0			0		
Systemic	159			156			2			1		
Insufficient information	24			24			0			0		
**Allergic vs not allergic AE**												
Allergic	1			1			0			0		
Not allergic	182			180			1			1		
Doubtful	26			25			1			0		
Insufficient information	3			3			0			0		
**Type of reporter**												
Self-reported	97			95			1			1		
Health-care worker	115			114			1			0		

**Sex**												
Male	59	54634	108	58	54050	107.3	1	565	177	0	19	0
Female	153	70221	217.9	151	69051	218.7	1	1039	96.2	1	131	763.4
**Age**												
<50	89	29154	305.3	89	28995	306.9	0	127	0	0	32	0
50-59	55	20230	271.9	54	20057	269.2	0	141	0	1	32	3125
60-69	29	22953	126.3	28	22689	123.4	1	240	416.7	0	24	0
70-79	10	17481	57.2	9	16437	54.8	1	1012	98.8	0	32	0
80+	29	35034	82.8	29	34923	83	0	81	0	0	30	0
**Vaccination motive**												
Population	52	54724	95	51	54181	94.1	1	475	210.5	0	68	0
Comorbidity	57	45043	126.5	56	43967	127.4	1	1027	97.4	0	49	0
Long-term care facilities (LTCF)	4	3123	128.1	4	3122	128.1	0	1	0	0	0	-
Work	99	21962	450.8	98	21831	448.9	0	98	0	1	33	3030.3

Table S3. Characteristics of adverse events after the first dose of AstraZeneca and J&J/Janssen COVID-19 vaccine.

	I Dose											
	Total			Green pathway			Yellow pathway without allergology visit			Percorso giallo con visita allergologica		
	N of AEs	N of vaccinated	Incidence	N of AEs	N of vaccinated	Incidence	N of AEs	N of vaccinated	Incidence	N of AEs	N of vaccinated	Incidence
**Overall**	309	57204	540.2	309	57204	540.2	0	0	-	0	0	-
**Severity**												
Severe	26			26			0			0		
Other clinically relevant condition	*12*			*12*			*0*			*0*		
Death	*0*			*0*			*0*			*0*		
Severe or permanent disability	*0*			*0*			*0*			*0*		
(Prolonged) hospitalisation	*14*			*14*			*0*			*0*		
Life threatening condition	*0*			*0*			*0*			*0*		
Not severe	283			283			0			0		
**Onset time**												
Immediate (≤ 24h)	262			262			0			0		
Non-immediate (> 24h)	47			47			0			0		
**Local vs systemic AE**												
Local	7			7			0			0		
Systemic	286			286			0			0		
Insufficient information	16			16			0			0		
**Allergic vs not AE**												
Allergic	1			1			0			0		
Not allergic	301			301			0			0		
Doubtful	7			7			0			0		
Insufficient information	0			0			0			0		
**Type of reporter**												
Self-reported	184			184			0			0		
Health-care worker	125			125			0			0		

**Sex**												
Male	81	24249	334	81	24249	334	0	0	-	0	0	-
Female	228	32955	691.9	228	32955	691.9	0	0	-	0	0	-
**Age**												
<50	130	8695	1495.1	130	8695	1495.1	0	0	-	0	0	-
50-59	49	4614	1062	49	4614	1062	0	0	-	0	0	-
60-69	55	18148	303.1	55	18148	303.1	0	0	-	0	0	-
70-79	75	25693	291.9	75	25693	291.9	0	0	-	0	0	-
80+	0	54	0	0	54	0	0	0	-	0	0	-
**Vaccination motive**												
Population	132	42987	307.1	132	42987	307.1	0	0	-	0	0	-
Comorbidity	1	306	326.8	1	306	326.8	0	0	-	0	0	-
Long-term care facilities (LTCF)	0	25	0	0	25	0	0	0	-	0	0	-
Work	176	13886	1267.5	176	13886	1267.5	0	0	-	0	0	-

Table S4. Characteristics of adverse events after the second dose of Pfizer/Moderna COVID-19 vaccine.

	II Dose														
	Total			Green pathway						Yellow pathway					
				Reaction to I dose			No reaction to I dose			Reaction to I dose			No reaction to I dose		
	N of AE	N vaccinated	Incidence	N of AE	N vaccinated	Incidence	N of AE	N vaccinated	Incidence	N of AE	N vaccinated	Incidence	N of AE	N vaccinated	Incidence
**Overall**	294	116738	251.8	3	34	8823.5	287	115020	249.5	0	1	0.0	4	1683	237.7
**Severity**															
Severe	24			0			23			0			1		
Other clinically relevant condition	*10*			*0*			*10*			*0*			*0*		
Death	*4*			*0*			*4*			*0*			*0*		
Severe or permanent disability	*1*			*0*			*1*			*0*			*0*		
(Prolonged) hospitalisation	*5*			*0*			*5*			*0*			*0*		
Life threatening condition	*4*			*0*			*3*			*0*			*1*		
Not severe	270			3			264			0			3		
**Onset time**															
Immediate (≤ 24h)	253			2			*247*			0			4		
Non-immediate (> 24h)	41			1			*40*			0			0		
**Local vs systemic AE**															
Local	13			0			*13*			0			0		
Systemic	260			3			*253*			0			4		
Insufficient information	21			0			*21*			0			0		
**Allergic vs not allergic reactions**															
Allergic	4			0			*4*			0			0		
Not allergic	276			3			270			0			3		
Doubtful	10			0			*9*			0			1		
Insufficient information	4			0			4			0			0		
**Type of reporter**															

Self-reported	123			0			122			0			1		
Health-care worker	171			3			165			0			3		
**Sex**															
Male	73	51144	142.7	0	2	0.0	71	50579	140.4	0	0	-	2	563	355.2
Female	221	65594	336.9	3	32	9375.0	216	64441	335.2	0	1	0.0	2	1120	178.6
**Age**															
<50	156	26919	579.5	1	13	7692.3	154	26759	575.5	0	0	-	1	147	680.3
50-59	67	18746	357.4	2	12	16666.7	63	18573	339.2	0	1	0.0	2	160	1250.0
60-69	29	21358	135.8	0	2	0.0	29	21106	137.4	0	0	-	0	250	0.0
70-79	16	16022	99.9	0	4	0.0	15	14994	100.0	0	0	-	1	1024	97.7
80+	26	33693	77.2	0	3	0.0	26	33588	77.4	0	0	-	0	102	0.0
**Vaccination motive**															
Population	48	52688	91.1	0	7	0.0	47	52165	90.1	0	0	-	1	516	193.8
Comorbidity	70	40784	171.6	0	4	0.0	69	39733	173.7	0	0	-	1	1047	95.5
Long-term care facilities (LTCF)	2	2644	75.6	0	0	-	2	2643	75.7	0	0	-	0	1	0.0
Work	174	20622	843.8	3	23	13043.5	169	20479	825.2	0	1	0.0	2	119	1680.7

Table S5. Characteristics of adverse events after the second dose of AstraZeneca and J&J/Janssen COVID-19 vaccine.

	**II Dose**														
	**Total**			**Green pathway**						**Yellow pathway**					
				**Reaction to I dose**			**No reaction to I dose**			**Reaction to I dose**			**No reaction to I dose**		
	**N of AE**	**N vaccinated**	**Incidence**	**N of AE**	**N vaccinated**	**Incidence**	**N of AE**	**N vaccinated**	**Incidence**	**N of AE**	**N vaccinated**	**Incidence**	**N of AE**	**N vaccinated**	**Incidence**
**Overall**	28	52453	53.4	0	5	0.0	28	52448	53.4	0	0	-	0	0	-
**Severity**															
Severe	3			0			3			0			0		
Other clinically relevant condition	*1*			*0*			*1*			*0*			*0*		
Death	*0*			*0*			*0*			*0*			*0*		
Severe or permanent disability	*0*			*0*			*0*			*0*			*0*		
(Prolonged) hospitalisation	*0*			*0*			*0*			*0*			*0*		
Life threatening condition	*2*			*0*			*2*			*0*			*0*		
Not severe	25			0			25			0			0		
**Onset time**															
Immediate (≤ 24h)	20			0			20			0			0		
Non-immediate (> 24h)	8			0			8			0			0		
**Local vs systemic AE**															
Local	1			0			1			0			0		
Systemic	24			0			24			0			0		
Insufficient information	3			0			3			0			0		
**Onset time**															
Allergic	0			0			0			0			0		
Not allergic	26			0			26			0			0		
Doubtful	2			0			2			0			0		
Insufficient information	0			0			0			0			0		
**Type of reporter**															

Self-reported	10			0			10			0			0		
Health-care worker	18			0			18			0			0		
**Sex**															
Male	9	22086	40.7	0	1	0.0	9	22085	40.8	0	0	-	0	0	-
Female	19	30367	62.6	0	4	0.0	19	30363	62.6	0	0	-	0	0	-
**Age**												-			-
<50	6	8225	72.9	0	2	0.0	6	8223	73.0	0	0	-	0	0	-
50-59	6	4348	138.0	0	1	0.0	6	4347	138.0	0	0	-	0	0	-
60-69	10	15017	66.6	0	0	-	10	15017	66.6	0	0	-	0	0	-
70-79	6	24821	24.2	0	2	0.0	6	24819	24.2	0	0	-	0	0	-
80+	0	42	0.0	0	0	-	0	42	0.0	0	0	-	0	0	-
**Vaccination motive**												-			-
Population	15	38964	38.5	0	2	0.0	15	38962	38.5	0	0	-	0	0	-
Comorbidity	1	248	403.2	0	0	-	1	248	403.2	0	0	-	0	0	-
Long-term care facilities (LTCF)	0	25	0.0	0	0	-	0	25	0.0	0	0	-	0	0	-
Work	12	13219	90.8	0	3	0.0	12	13216	90.8	0	0	-	0	0	-
